# The impact of emotion awareness and regulation on psychotic symptoms during daily functioning

**DOI:** 10.1038/s41537-020-0096-6

**Published:** 2020-03-24

**Authors:** David Kimhy, Amanda Lister, Ying Liu, Julia Vakhrusheva, Philippe Delespaul, Dolores Malaspina, Luz H. Ospina, Vijay A. Mittal, James J. Gross, Yuanjia Wang

**Affiliations:** 1 Department of Psychiatry, Icahn School of Medicine at Mount Sinai, New York, NY USA; 2 MIRECC, James J. Peters VA Medical Center, Bronx, NY USA; 3 New York State Psychiatric Institute, New York, NY USA; 4 Department of Psychiatry, Columbia University, New York, NY USA; 5 Departments of Psychiatry & Neuropsychology, Maastricht University, Maastricht, The Netherlands; 6 Department of Psychology, Northwestern University, Chicago, IL USA; 7 Department of Psychology, Stanford University, Stanford, CA USA

**Keywords:** Human behaviour, Schizophrenia

## Abstract

Emotion regulation (ER) difficulties are ubiquitous among individuals with schizophrenia and have been hypothesized to contribute to stress sensitivity and exacerbation of psychotic symptoms in this population. However, the evidence supporting this link is equivocal, potentially due to previous studies’ reliance on retrospective assessments of ER and psychosis, as well as lack of consideration of putative moderators such as emotion awareness. To address these limitations, we employed experience sampling method using mobile electronic devices to investigate the links between momentary in vivo use of ER strategies (_m_ER), emotion awareness, and psychotic symptoms during daily functioning. Fifty-four individuals with schizophrenia completed assessment of _m_ER and psychotic symptoms, along with traditional retrospective measures of ER and symptoms. Use of _m_ER suppression predicted significant increases in momentary experiences of thought insertion, mind reading, auditory and visual hallucinations. Use of _m_ER reappraisal predicted significant increases in momentary experiences of suspiciousness, thought insertion, and mind reading. Emotion awareness, driven primarily by difficulties identifying feelings, moderated the impact of ER on psychotic symptoms. There were no associations between retrospective measures of ER and symptoms. Our results indicate that, among individuals with schizophrenia, emotion awareness significantly impacts the relationship between use of ER and exacerbations in psychotic symptoms during the course of daily functioning. Our results highlight the need to incorporate emotion awareness and regulation difficulties into the development of treatment models and interventions for psychosis. In addition, our results underscore the need to employ in vivo, high time-resolution assessment methods to study dynamic clinical phenomena such as ER and psychotic symptoms.

## Introduction

Emotional difficulties are ubiquitous among individuals with schizophrenia and are considered a core feature of the disorder^1^. Specifically, individuals with schizophrenia display substantial emotion regulation (ER) difficulties that have been proposed to play a key role in both the initial onset of psychosis^2^, as well as exacerbations of psychotic symptoms^3–6^. Previous investigations have examined a wide range of ER strategies^7^. Among these, the process model proposed by Gross et al. has received the most empirical attention in the basic affective and clinical literatures^8,9^. The model highlights two primary ER strategies, characterized by the use of reappraisal (antecedent-focused) and suppression (response-focused) ER strategies^8^. Among nonclinical populations, use of reappraisal has been linked to enhanced social functioning, greater expression of positive emotion, lower negative emotional experience, and higher quality of life^10–12^. In contrast, use of suppression has been linked to poorer social functioning, lower social support, lower satisfaction and closeness to others, greater expression of negative emotion, as well as decreased well-being^8,13,14^.

Germane to schizophrenia, previous reports have documented increased use of suppression and more limited use of reappraisal among individuals with psychotic spectrum disorders including in individuals with schizophrenia^7,15,16^, at clinical high-risk for psychosis^16,17^, as well as in nonclinical psychosis-prone individuals^16,18^. A recent large systematic review and meta-analysis incorporating data from 42 studies (2498 individuals with psychosis and 3381 healthy controls) provided strong support for these associations^19^. Yet, evidence from studies examining the impact of ER on psychotic symptoms points to a more equivocal link. An analysis of the association between use of adaptive ER strategies (e.g., cognitive reappraisal, acceptance, and awareness; six studies) and psychosis found a nonsignificant effect, with moderate to high degree of heterogeneity in outcomes^19^. Notably, the majority of studies examined overall psychosis burden rather than individual psychotic symptoms, with only one study reporting an association with a specific symptom, paranoia^20^. An examination of the link between use of maladaptive ER strategies (e.g., suppression, self-blaming, rumination) and psychosis found a small to moderate effect, but with moderate to high heterogeneity in outcomes. Removal of the only study examining a specific symptom^20^ from this meta-analysis resulted in a smaller effect.

A number of factors may potentially contribute to the heterogeneity in findings—one critical factor may have been previous reports’ reliance on retrospective assessments of ER and symptoms. Such assessments are vulnerable to the influence of episodic memory difficulties, emotional states at the time of the assessment, as well as cognitive biases and reframing^21,22^. This issue is particularly acute in studies of individuals with schizophrenia given the substantial episodic memory deficits documented in this population^23,24^. Consistent with these findings, poor memory has been found to have a detrimental impact on accuracy of recollected mood and symptoms among individuals with schizophreia^25^. Another factor may be the fact that a plurality of the studies reviewed by Ludwig et al. examined the impact of ER on total psychosis burden, rather than individual symptoms, potentially obscuring granular ER influences on specific symptoms^19^. Finally, many individuals with schizophrenia display poor emotion awareness, characterized by difficulties identifying, labeling, and/or differentiating their own emotions^15,17^. The presence of such difficulties may have a detrimental effect on ER effectiveness and obfuscate the ability to identify potential affective processes underlying the impact of ER on psychotic symptoms. While the terms emotion awareness, ER, and alexithymia share some conceptual overlap, they also represent distinct clinical phenomena. Specifically, while poor emotion awareness is a central component of alexithymia, the latter also incorporates cognitive aspects (i.e., externally oriented thinking, EOT). Likewise, implicit to the process of ER is the need to accurately identify emerging emotions in self in order to employ effective ER strategies.

To address some of the limitations associated with use of retrospective assessments, researchers have employed experience sampling method (ESM), an ecologically valid, time-sampling method of self-reports developed to study the dynamic process of person–environment interactions^26^ that has been used extensively to study psychosis^26^. A number of authors have used ESM to investigate the links between ER and psychosis—Visser et al. examined ER in 28 individuals with schizophrenia and 28 demographically matched healthy controls and found adequate ER effort in the schizophrenia group, but poor effectiveness^27^. Specifically, the schizophrenia group initiated ER efforts at a lower negative emotion threshold; they selected more strategies than the control group and those strategies were less contextually appropriate; and when employing moderate to high levels of effort, they were less effective at decreasing negative emotions. In a separate data analysis using the same schizophrenia sample, Strauss et al. found that participants tried to implement ER strategies frequently during psychotic experiences, but those attempts were ineffective at reducing negative emotion^28^. Furthermore, patients who had less effective ER during psychotic experiences had more densely connected individual emotions^28^, echoing earlier findings of low emotional granularity among individuals with schizophrenia^29^. Finally, Nittel et al. examined 32 individuals with psychosis and found that use of suppression in response to negative emotions was associated with increases in paranoia during daily functioning^30^. Yet, these ESM studies had a number of limitations—Visser et al. used ESM to assess ER, however psychotic symptoms were evaluated using retrospective measures^27^. Nittel et al. employed a robust ESM design to assess both ER and symptoms; however, their examination was rather narrow, focusing solely on paranoia^30^. Strauss et al. used ESM to concurrently assess both ER and a range of individual symptoms^28^. However, the impact of emotion awareness on ER and psychotic symptoms was not examined. Hence, the putative influence of this factor remains unknown.

To address these limitations, we sought to explore the associations between ER, emotion awareness, and a range of psychotic symptoms in individuals with schizophrenia using ambulatory, momentary “in vivo, in situ” assessments during the course of daily functioning. Specifically, we aim to (1) confirm previous findings regarding the associations of retrospective assessments of ER and symptoms. Then, employing ESM approach, we aimed to examine (2) the impact of momentary, in vivo use of suppression and reappraisal ER strategies (_m_ER) on psychotic symptoms during “real-world” daily functioning; and (3) whether emotion awareness moderated the impact of ER on changes in psychotic symptoms.

## Results

Data were collected on 54 individuals with schizophrenia and related disorders (41 schizophrenia, 11 schizoaffective disorder, and 2 schizophreniform disorder). The sample’s average age was 32.31 (SD = 8.23) and had an average 14.73 years of formal education (SD = 3.48). Sixty percent were female and 89% were never married. The sample was racially and ethnically diverse—52% were Caucasian, 15% Black/African-American, 11% Asian, and 22% more than one race. Twenty-nine percent were Hispanic. The participants’ average reading ability, as indexed by WTAR total score, was 39.61 (SD = 8.42; potential range 0–50). The average dosage of prescribed antipsychotic medication was 323.79 (SD = 335.42), as indexed by chlorpromazine equivalence. Age was not associated with use of ER strategies or severity of psychotic symptoms. Participants responded on average to 15.07 (SD = 4.12) experience samples over the assessment period (75.4% response rate), consistent with previous ESM studies of people schizophrenia^22,31–34^.

**Fig. 1:**
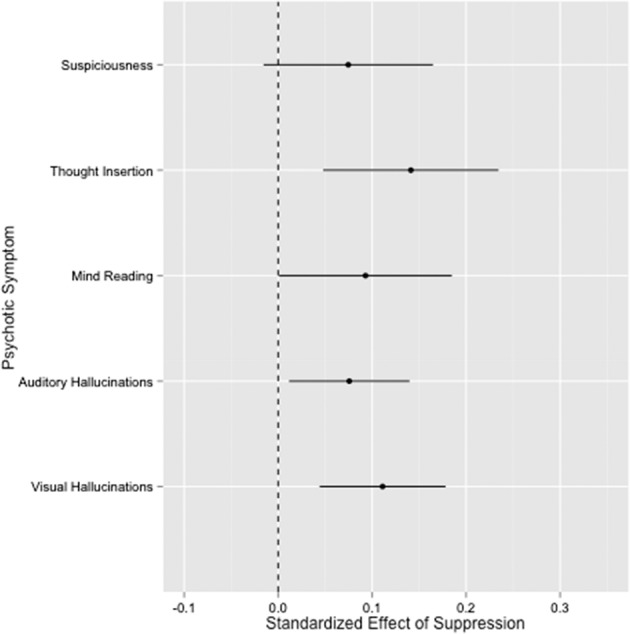
Effect sizes of the impact of use of suppression on psychotic symptoms in individuals with schizophrenia.

Our first aim was to examine the links between traditional retrospective measures of ER and psychotic symptoms—there were no significant associations between Emotion Regulation Questionnaire (ERQ) suppression and reappraisal and any of the Scale for the Assessment of Positive Symptoms (SAPS)-indexed psychotic symptoms. In contrast, employment of _m_ER suppression and _m_ER reappraisal strategies during the course of daily functioning resulted in significant impact on psychotic symptoms. The standardized effects representing links between use of _m_ER strategies and psychotic symptoms are presented in Table 1. Specifically, use of _m_ER suppression predicted momentary exacerbations in experiences of thought insertion, mind reading, as well as auditory and visual hallucinations (see Fig. 1). Similarly, use of _m_ER reappraisal predicted significant increases in suspiciousness, thought insertion, and mind reading.

**Table 1:** The impact of emotion regulation strategies on psychotic symptoms during the course of daily functioning in individuals with schizophrenia.

Psychotic symptom	Emotion regulation strategy	Estimate	SE	*T* value	*p*
Suspiciousness	Reappraisal	0.10	0.04	2.21	**0.03**
	Suppression	0.07	0.05	1.62	0.11
Thought insertion	Reappraisal	0.26	0.04	5.99	**<0.0001**
	Suppression	0.14	0.05	2.97	**0.0032**
Mind reading	Reappraisal	0.22	0.04	5.02	**<0.0001**
	Suppression	0.09	0.05	1.98	**0.05**
Auditory hallucinations	Reappraisal	−0.01	0.03	−0.31	0.76
	Suppression	0.08	0.03	2.31	**0.02**
Visual hallucinations	Reappraisal	0.02	0.03	0.50	0.62
	Suppression	0.11	0.03	3.25	**<0.01**

*N* = 54. Significant associations are marked in bold.

**Fig. 2:**
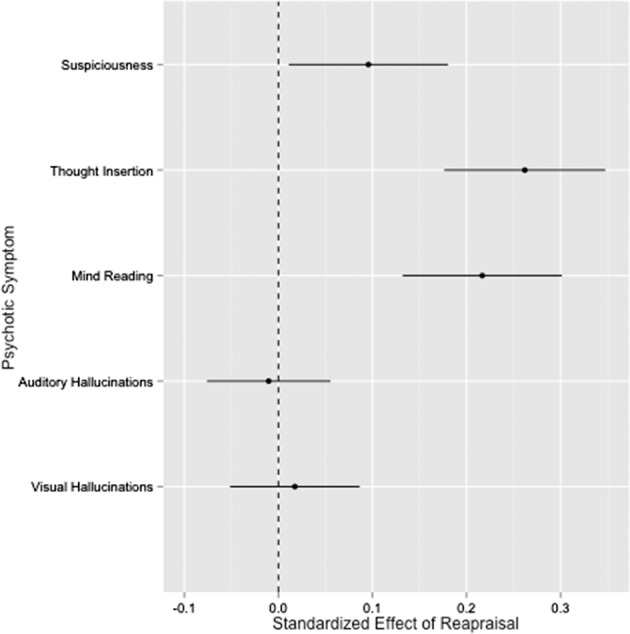
Effect sizes of the impact of use of cognitive reappraisal on psychotic symptoms in individuals with schizophrenia.

Finally, we explored whether emotion awareness moderated the impact of momentary use of _m_ER strategies on changes in psychotic symptoms. Poor emotion awareness, as indexed by TAS-20 Total Score, had a significant moderating effect on the associations between _m_ER suppression, and thought insertion and mind reading (standardized effect = 0.01, *p* < 0.01 and standardized effect = 0.01, *p* < 0.0001, respectively). Specifically, use of _m_ER suppression predicted increases in thought insertion and mind reading more strongly in individuals with poorer emotion awareness. Moderating effects were also observed for the association between _m_ER reappraisal and paranoia (standardized effect = −0.16, *p* < 0.001), with reappraisal predicting increases in paranoia more strongly in individuals with better emotion awareness. The impact of emotion awareness on _m_ER was driven primarily by difficulties identifying feelings (DIF), which displayed moderating effects on the association between _m_ER suppression and mind reading and visual hallucinations (standardized effect = 0.02, *p* = 0.001 and standardized effect = 0.01, *p* = 0.003, respectively), as well as reappraisal and paranoia (standardized effect = −0.18, *p* < 0.0001). In contrast, the TAS-20 domain of difficulties describing feelings (DDF) did not have a significant impact on the association between use of _m_ER strategies and psychotic symptoms (Fig. 2).

## Discussion

To the best of our knowledge, the present investigation is the first study to characterize the momentary in vivo links between use of ER strategies, poor emotion awareness, and psychotic symptoms during the course of real-world daily functioning in individuals with schizophrenia. The most important findings are as follows: (1) the confirmation of temporal links between momentary use of ER strategies and transitory changes in severity of psychotic symptoms; and (2) the identification of poor emotion awareness as an important moderator of the association between ER on psychotic symptoms. Specifically, use of suppression by individuals with schizophrenia predicted momentary elevations in experiences of thought insertion, mind reading, and auditory and visual hallucinations. These results are consistent with findings by Strauss et al.^28^, as well as previous reports employing retrospective measures that identified links between ERQ-indexed use of suppression and more severe auditory hallucinations (as indexed by the Psychotic Symptom Rating Scales, PSYRATS)^3^. We did not find such associations using retrospective measures of suppression and psychotic symptoms (ERQ and SAPS), potentially due to the use of a less sensitive measure of psychosis (SAPS vs. PSYRATS).

Similarly, momentary in vivo use of reappraisal predicted episodic elevations in suspiciousness, thought insertion, and mind reading. These results are somewhat surprising, given previous reports associating reappraisal use with enhanced functioning. One possible explanation may be related to the distinction between the employment of reappraisal strategies vs. their actual effectiveness. While individuals with schizophrenia have been shown to be able to use reappraisal when instructed to do so in laboratory settings and such effort lead to down regulation of anxiety^20,35^, results from real-world studies employing mobile assessments support the view of adequate ER effort, but poor effectiveness^27^. Specifically, individuals with schizophrenia have been observed to initiate ER efforts at a lower threshold of negative emotion intensity compared to healthy controls^27^. They also tended to use more ER strategies, yet the attempted strategies were less contextually appropriate, and despite moderate to high levels of effort, use of such strategies was ineffective at decreasing negative emotion^27^. These results are also consistent with reports from investigations utilizing event-related potentials indicating such efforts to regulate emotion are often ineffective^36–38^. The discrepancy in the results may be related to differences between use of self-reports in the laboratory vs. physiological measures. In addition, assessments conducted in “real-world” settings may potentially present substantially greater level of environmental stimulation, cognitive load, and stress vs. the more controlled environments of laboratories. Alternatively, our results may relate to our sample’s characteristics—participants recruited to this study had moderate or more severe positive symptoms (score of ≥3 on the SAPS hallucinations/delusions items). Thus, the limited effectiveness of use of reappraisal may reflect the more acute character of our sample. Laboratory studies of healthy individuals have found that presentation of higher intensity emotional stimuli have been linked to employment of non-reappraisal ER strategies^39^. Perhaps individuals with schizophrenia with less severe and/or less frequent symptoms may display a more effective use of reappraisal. Future studies should aim to examine this issue.

Our findings of emotion awareness moderating the effect of ER on psychotic symptoms are intriguing and are consistent with previous reports that linked poor emotion awareness, a central element of alexithymia, to psychotic symptoms in schizophrenia^40,41^ and risk for psychosis^42,43^. This perspective is also in agreement with the broader conceptualization of alexithymia as a disorder of affect regulation^44^. Poor emotion awareness has been found to be related to severity of hallucinations, but not delusions, in studies employing retrospective measures, with patients with a lifetime history of more psychotic symptoms displaying poorer emotion awareness^45^. In addition, poor emotion awareness has been found to be a major predictor of social functioning deficits in individuals with schizophrenia^15,46^ and at-risk for psychosis^17^.

**Fig. 3:**
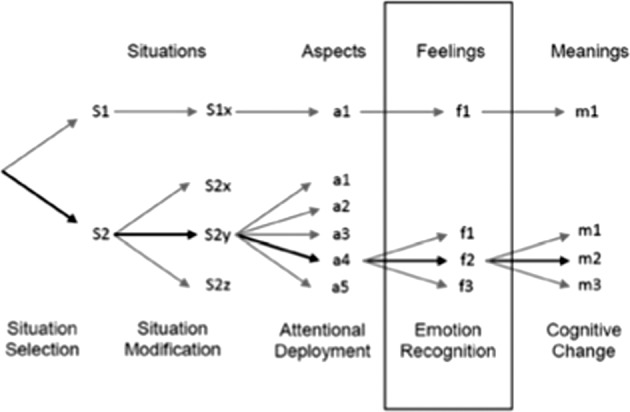
An adapted model of Gross’ Emotion Regulation Process (Moyal et al.^47^; adapted with permission). Moyal et al.^47^ expanded on Gross’s originalmodel of emotion regulation to include emotion labeling as a critical process underlying the effectiveness of emotion regulation. Specifically, the adapted model posits that accurate labeling of emotions is an essential process underlying effective use reappraisal emotion regulation strategy.

Our findings are consistent with the work of Moyal et al.^47^, who expanded on Gross’s original ER model to include emotion labeling (see Fig. 3) as a critical phase underlying effectiveness of ER. Specifically, Moyal et al. stated that difficulties labeling emotions might impact the effectiveness of use of reappraisal in clinical populations with deficits in emotion awareness, noting “in order to adaptively reappraise a situation and down-regulate a negative emotion, one has to attend to one’s feelings and understand them, and not only appraise the situation in a general way” (ref. ^47^, p. 3). Consistent with this view, Strauss et al. found that ER difficulties in individuals with schizophrenia were not related to the identification of whether negative emotions are present, but rather to difficulties selecting and implementing appropriate ER strategies^28^. As coping with different emotions may call for use of distinct ER strategies^29^, such selection is dependent, in part, on the ability to accurately read and interpret internal affective state signals. Thus, individuals with schizophrenia may be able to identify the presence of negative states in general, but their ability to read and label affective signals in a granular way may be compromised, potentially impacting the effectiveness of their ER efforts. Future studies should aim to further elucidate the mechanism underlying poor emotion awareness among individuals with schizophrenia and at-risk for psychosis and delineate their impact on ER and symptoms.

Our results highlight the relevance of poor emotion awareness and ER for the treatment of individuals with schizophrenia and need to incorporate emotion awareness and regulation difficulties into the development of treatment models and interventions for psychosis. While ER skills are important components of many contemporary psychotherapy approaches including ACT, DBT, as well as emotion-focused therapy^48,49^, emotion awareness deficits have received relatively little attention in the treatment of schizophrenia. Specifically, CBT for psychosis treatment models for this population have emphasized cognitive difficulties far more than emotional ones. However, given recent findings by our group and others^27,28,30^, poor emotion awareness and ER difficulties should receive more clinical attention in the development and refinement of future treatment models of psychosis in schizophrenia.

The present study has a number of strengths including being the largest study to date to investigate ER and psychosis using an ESM approach (*n* = 54), availability of a sample with high degree of racial and ethnic diversity, employment of ESM to concurrently to evaluate both ER and psychotic symptoms, as well as use of state-of-the-art assessments of diagnoses and symptoms. Notably, the present study has some limitations as well. First, only two ER strategies were examined. Future studies should investigate additional strategies and their potential impact on symptoms. Second, the momentary use of in vivo ER and psychotic symptoms were assessed in the same experience samples. Thus, while unlikely, we could not exclude the possibility that the reported associations were influenced by the participants’ mood at the moment or another concurrent clinical variable. Third, emotion awareness was evaluated using a trait scale, rather than via ESM. Finally, we did not assess episodic memory, thus we could not control for the potential impact of memory deficits on the outcomes. Previous reports indicated significant correlations between in vivo and retrospective measures of depressed mood among individuals with schizophrenia^25^. However, once variance due to memory was statistically controlled, the relationship between the in vivo and retrospective measures was no longer significant. Thus, while participants were asked to recall very recent experiences (up to ~1½ hours earlier), the potential of such biases impacting outcome variables is possible.

In summary, the present investigation is the first study to characterize in vivo and in situ the links between use of ER strategies, emotion awareness, and psychotic symptoms during the course of daily functioning in individuals with schizophrenia. The results extend previous findings and highlight the substantial ER difficulties individuals with schizophrenia experience during daily functioning and the key role poor emotion awareness play in contributing to such difficulties. Our results indicate that these deficits have a substantial impact on the ebb-and-flow of psychotic symptoms during daily functioning. Our results also highlight the importance of employing high time-resolution assessment methods to investigate dynamic clinical phenomena such as ER and psychotic symptoms.

## Methods

### Participants

Analyses used data obtained from baseline assessments of two affiliated studies (*n* = 16 and *n* = 38) conducted at the New York State Psychiatric Institute (NYSPI) that examined the links between changes in ambulatory measures of psychotic symptoms, autonomic functioning, and ER. The two studies were supported by a single grant from the National Institute of Mental Health (NIMH; PI: DK, K23 MH077653). There were no significant differences between participants in the two studies with regard to age, ethnicity, marital status, years of education, or reading ability. Study 1 (*n* = 16) had a higher proportion of Caucasian participants, but this characteristic was not associated with not associated with the primary variables of interest. The data reported in this manuscript were collected as part of the studies baseline assessments, which employed identical measures and data collection procedures. Participants for both studies were recruited from patients treated at the same medical center and data collection was in parallel.

Data collection procedures and measures used in the baseline assessments of both studies were identical, with data collection taking place concurrently between June 2008 and May 2013. Participants for both studies were recruited from patients treated at the NYSPI. The inclusion criteria were ages 18–55; English speaking; IQ > 80; a DSM-IV diagnosis of schizophrenia or related disorders (schizophrenia, schizoaffective disorder, and schizophreniform disorder); presence of moderate or more severe hallucinations and/or delusions (≥3 on SAPS); and being able to provide informed consent. The exclusion criteria were recent use of street drugs (confirmed via a urine toxicology test); low reading ability (WTAR < 7); and history of severe cardiac conditions assessed via self-report (the studies included assessments of cardiac autonomic regulation not covered in the present manuscript). The NYSPI Institutional Review Board approved the study and all subjects provided written informed consent.

### Procedures

Participants satisfying the inclusion and exclusion criteria completed the baseline research assessment, which included diagnostic, symptom, emotion awareness and ER evaluations, typically within 2–3 weeks of admission to the study. The ambulatory assessments of _m_ER strategies and psychotic symptoms were conducted during weekdays over a 36-hour period (10 am day 1 → 10 pm day 2) using ESM with mobile electronic devices (Palm Tungsten T^3^). As part of the protocol, participants also wore a vest-like undergarment embedded with sensors designed to record ambulatory cardiopulmonary autonomic data (not reported in the present manuscript). A 36-hour assessment period was selected in consideration of the participants’ comfort level—participants could not take showers during this period, as it would have required them to disconnect and reattach the ECG electrodes by themselves^22,34^.

On the morning of the ambulatory assessment, participants received a brief introductory session on operation of the mobile devices and were provided with a mobile device to carry throughout the ambulatory assessment. Dedicated software (iESP, Intel Corporation, Seattle, WA) was used to present questions and collect responses on the mobile devices. The software was pre-programmed to beep 10 times/day (10 am → 10 pm) at random times to elicit self-reports. Upon hearing the beep, participants were instructed to report the presence of any current psychotic symptoms; a recent stressor since the previous beep (which typically occurred up to ~1½ hours earlier); and the degree to which they used particular ER strategies to address this stressor. Stressors were defined as any thoughts or events (major and/or minor) resulting in increase in stress. Responses were represented in the output as a value between 1 (“not at all”; left-most extreme) and 100 (“very much”; right-most extreme).

### Measures

Diagnoses were determined using the Diagnostic Interview for Genetic Studies (DIGS)^50^, a clinical interview developed for the assessment of major mood and psychotic disorders and their spectrum conditions. The DIGS was developed as a collaborative effort of investigators from sites within the NIMH Genetics Initiative. Test–retest (within-site, between-site) reliability for DSM-III-R criteria-based major depression, bipolar disorder, schizophrenia, and schizoaffective disorder were excellent (0.73–0.95).

Psychotic symptoms were assessed using retrospective measures and momentary in vivo evaluations. Retrospective interview-based assessments of psychotic symptoms during the previous week were conducted using SAPS, a semi-structured interview^51^. In vivo assessments of psychotic symptoms were conducted using mobile devices employing an ESM approach—our group and others have published extensively on use of this methodology among individuals with schizophrenia^22,27,28,30–34^. Assessment of momentary in vivo psychotic symptoms included suspiciousness, thought insertion, mind reading, and auditory and visual hallucinations.

Similarly, evaluations of ER were conducted using retrospective and momentary in vivo measures. The ERQ^13^, a 10-item self-report questionnaire was used to assess global ER. The ERQ includes six items assessing reappraisal and four assessing suppression. Participants are asked to indicate on a seven-point scale (from 1 = “strongly disagree” to 7 = “strongly agree”) to what extent they agree with each statement, with higher scores reflecting stronger endorsement of the strategy. The ERQ is a valid and reliable measure of ER, with an average alpha reliability of 0.79 for reappraisal and 0.73 for suppression. Test–retest correlations across three months were 0.69 for both scales^13^. Momentary in vivo assessments of ER during daily functioning (_m_ER) were conducted using mobile devices employing ESM. To maintain fidelity with the ER strategies of the ERQ, we adapted four items from the ERQ (2 reappraisal, 2 suppression) to index _m_ER. The average score of the items “I keep my emotions to myself” and “I was careful not to express my emotions” was used to index _m_ER suppression. The average score of the items “I made myself think about it in a way that helped me stay calm” and “I controlled my emotions by changing the way I was thinking about the experience” was used to index _m_ER reappraisal.

Emotion awareness was assessed using the Toronto Alexithymia Scale (TAS-20)^52,53^, a 20-item self-report questionnaire. The TAS-20 has three subscales: DIF (7 items), DDF (5 items), and EOT (8 items). Participants are asked to indicate on a 5-point scale (from 1 = “strongly disagree” to 5 = “strongly agree”) to what extent they agree with each statement, with higher scores indicating more severe psychopathology. The TAS-20 has a good internal consistency (≥0.80) with the DIF and DDF subscales demonstrating solid reliability (*r* = 0.79–0.83). We elected to exclude from analyses the EOT subscale due to questionable reliability^54^.

Demographic and clinical information were collected including age, sex, race, ethnicity, medication use, as well as ratings of depression and anxiety (Beck Depression and Anxiety Inventories). In addition, we evaluated reading proficiency (Wechsler adult Test of Reading, WTAR) to ensure participants’ ability to read and comprehend texts presented on the questionnaires and ESM probes.

### Data analyses

In accordance with standards used in previous ESM schizophrenia studies^22,26,31,34^, participant data were excluded from analysis if they completed fewer than 33% of the ESM samples. Associations between retrospective measures of ER and symptoms were analyzed using Pearson correlations. The effect of momentary in vivo use of suppression and reappraisal on psychotic symptoms was assessed using linear mixed effects models with subject-specific random intercepts. The outcome was the momentary measurement of psychotic symptoms at time i, with _m_ER suppression_(i)_ or _m_ER reappraisal_(i)_ serving as the predictors of interest, adjusted for day effect. Finally, we examined the moderating effect of poor emotion awareness on the association between _m_ER suppression_(i)_ and _m_ER reappraisal_(i)_ on psychotic symptoms by including an interaction term in the linear mixed effects model. We analyzed separately the moderating effect of three aspects of alexithymia—the TAS-20 total score, and the DIF and DDF factors, with the Holm’s sequential Bonferroni procedure used to control for Type I error.

## Supplementary information

Reporting Sum

## Data Availability

All data are available upon request from the corresponding author.
