# Supplementary material for: The impact of emotion awareness and regulation on psychotic symptoms during daily functioning
Source: NPJ Schizophr. 2020 Mar 24;6:7. doi: 10.1038/s41537-020-0096-6 (PMC7093537; doi:10.1038/s41537-020-0096-6)
Supplement: Supplementary file 1 — Reporting Sum [file 41537_2020_96_MOESM1_ESM.pdf]

nature research

Corresponding author(s): Dr. David Kimhy

☐ Initial submission ☒ Revised version ☐ Final submission

Reporting Summary

Nature Research wishes to improve the reproducibility of the work that we publish. This form provides structure for consistency and transparency in reporting. For further information on Nature Research policies, see Authors & Referees and the Editorial Policy Checklist.

Please do not complete any field with "not applicable" or n/a. Refer to the help text for what text to use if an item is not relevant to your study.
For final submission: please carefully check your responses for accuracy; you will not be able to make changes later.

Statistical parameters

When statistical analyses are reported, confirm that the following items are present in the relevant location (e.g. figure legend, table legend, main text, or Methods section).

n/a	Confirmed	
☐	☒	The exact sample size (n) for each experimental group/condition, given as a discrete number and unit of measurement
☐	☒	An indication of whether measurements were taken from distinct samples or whether the same sample was measured repeatedly
☐	☒	The statistical test(s) used AND whether they are one- or two-sided<br>*Only common tests should be described solely by name; describe more complex techniques in the Methods section.*
☐	☒	A description of all covariates tested
☐	☒	A description of any assumptions or corrections, such as tests of normality and adjustment for multiple comparisons
☐	☒	A full description of the statistics including central tendency (e.g. means) or other basic estimates (e.g. regression coefficient) AND variation (e.g. standard deviation) or associated estimates of uncertainty (e.g. confidence intervals)
☐	☒	For null hypothesis testing, the test statistic (e.g. F, t, r) with confidence intervals, effect sizes, degrees of freedom and P value noted<br>*Give P values as exact values whenever suitable.*
☒	☐	For Bayesian analysis, information on the choice of priors and Markov chain Monte Carlo settings
☐	☒	For hierarchical and complex designs, identification of the appropriate level for tests and full reporting of outcomes
☒	☐	Estimates of effect sizes (e.g. Cohen's d, Pearson's r), indicating how they were calculated
☒	☐	Clearly defined error bars<br>*State explicitly what error bars represent (e.g. SD, SE, CI)*

Our web collection on statistics for biologists may be useful.

Software and code

Policy information about availability of computer code

Data collection	
Data analysis	https://github.com/dinhngockhanh/stochasticMRDinAML

For manuscripts utilizing custom algorithms or software that are central to the research but not yet described in published literature, software must be made available to editors/reviewers upon request. We strongly encourage code deposition in a community repository (e.g. GitHub). See the Nature Research guidelines for submitting code & software for further information.

Data

Policy information about availability of data

All manuscripts must include a data availability statement. This statement should provide the following information, where applicable:
- Accession codes, unique identifiers, or web links for publicly available datasets
- A list of figures that have associated raw data
- A description of any restrictions on data availability

Field-specific reporting

Please select the best fit for your research. If you are not sure, read the appropriate sections before making your selection.

☐ Life sciences ☒ Behavioural & social sciences

Life sciences

Study design

All studies must disclose on these points even when the disclosure is negative.

Sample size	54 individuals with schizophrenia and related disorders
Data exclusions	n/a
Replication	n/a
Randomization	n/a
Blinding	n/a

Materials & experimental systems

Policy information about availability of materials

n/a	Involved in the study
☒	☐ Unique materials
☒	☐ Antibodies
☒	☐ Eukaryotic cell lines
☒	☐ Research animals
☐	☒ Human research participants

We require information from authors about some experimental systems used in the life sciences. Here, indicate whether each material or system listed is relevant to your study.

If you are not sure if a material or system applies to your research, read the appropriate section below before selecting a response.

Unique materials

Obtaining unique materials

Antibodies

Antibodies used

Validation

Eukaryotic cell lines

Policy information about cell lines

Cell line source(s)

Authentication

Mycoplasma contamination

Commonly misidentified lines
(See ICLAC register)

Research animals

Policy information about studies involving animals; ARRIVE guidelines recommended for reporting animal research

Animals/animal-derived materials

Human research participants

Policy information about studies involving human research participants

Population characteristics	Individuals with schizophrenia and related disorders

Method-specific reporting

n/a	Involved in the study
☒	☐ ChIP-seq
☒	☐ Flow cytometry
☒	☐ Magnetic resonance imaging

We also require information from authors about specific methods used in the life sciences. Here, indicate whether each method listed is relevant to your study.

If you are not sure if a method applies to your research, read the appropriate section below before selecting a response.

ChIP-seq

Data deposition

☐ Confirm that both raw and final processed data have been deposited in a public database such as GEO.

☐ Confirm that you have deposited or provided access to graph files (e.g. BED files) for the called peaks.

Data access links<br>*May remain private before publication.*	
Files in database submission	
Genome browser session<br>(e.g. UCSC)	

Methodology

Replicates	
Sequencing depth	
Antibodies	
Peak calling parameters	
Data quality	
Software	

Flow Cytometry

Plots

Confirm that:

☐ The axis labels state the marker and fluorochrome used (e.g. CD4-FITC).

☐ The axis scales are clearly visible. Include numbers along axes only for bottom left plot of group (a 'group' is an analysis of identical markers).

☐ All plots are contour plots with outliers or pseudocolor plots.

☐ A numerical value for number of cells or percentage (with statistics) is provided.

Methodology

Sample preparation	
Instrument	
Software	
Cell population abundance	
Gating strategy	

☐ Tick this box to confirm that a figure exemplifying the gating strategy is provided in the Supplementary Information.

Magnetic resonance imaging

Experimental design

Design type

Design specifications

Behavioral performance measures

Acquisition

Imaging type(s)

Field strength

Sequence & imaging parameters

Area of acquisition

Diffusion MRI ☐ Used ☐ Not used

Preprocessing

Preprocessing software

Normalization

Normalization template

Noise and artifact removal

Volume censoring

Statistical modeling & inference

Model type and settings

Effect(s) tested

Specify type of analysis: ☐ Whole brain ☐ ROI-based ☐ Both

Statistic type for inference
(See Eklund et al. 2016)

Correction

Models & analysis

n/a	Involved in the study
☐	☐ Functional and/or effective connectivity
☐	☐ Graph analysis
☐	☐ Multivariate modeling or predictive analysis

Functional and/or effective connectivity

Graph analysis

Multivariate modeling and predictive analysis

Behavioural & social sciences

Study design

All studies must disclose on these points even when the disclosure is negative.

Study description A study employing Experience Sampling Method using mobile digital devices to investigate the links between momentary "

Research sample	54 individuals with schizophrenia and related disorders
Sampling strategy	n/a
Data collection	via clinical interviews and ambulatory assessment with mobile digital devices
Timing	June 2008 to May 2013
Data exclusions	n/a
Non-participation	n/a
Randomization	n/a

This checklist template is licensed under a Creative Commons Attribution 4.0 International License, which permits use, sharing, adaptation, distribution and reproduction in any medium or format, as long as you give appropriate credit to the original author(s) and the source, provide a link to the Creative Commons license, and indicate if changes were made. The images or other third party material in this article are included in the article's Creative Commons license, unless indicated otherwise in a credit line to the material. If material is not included in the article's Creative Commons license and your intended use is not permitted by statutory regulation or exceeds the permitted use, you will need to obtain permission directly from the copyright holder. To view a copy of this license, visit http://creativecommons.org/licenses/by/4.0/
